# A practical approach to Sasang constitutional diagnosis using vocal features

**DOI:** 10.1186/1472-6882-13-307

**Published:** 2013-11-07

**Authors:** Jun-Su Jang, Boncho Ku, Young-Su Kim, Jiho Nam, Keun Ho Kim, Jong Yeol Kim

**Affiliations:** 1 Medical Engineering R&D Group, Medical Research Division, Korea Institute of Oriental Medicine, 1672 Yuseongdae-ro, Yuseong-gu, Daejeon 305-811, Republic of Korea

**Keywords:** Sasang constitution, Diagnosis, Voice, Vocal feature

## Abstract

**Background:**

Sasang constitutional medicine (SCM) is a type of tailored medicine that divides human beings into four Sasang constitutional (SC) types. Diagnosis of SC types is crucial to proper treatment in SCM. Voice characteristics have been used as an essential clue for diagnosing SC types. In the past, many studies tried to extract quantitative vocal features to make diagnosis models; however, these studies were flawed by limited data collected from one or a few sites, long recording time, and low accuracy. We propose a practical diagnosis model having only a few variables, which decreases model complexity. This in turn, makes our model appropriate for clinical applications.

**Methods:**

A total of 2,341 participants’ voice recordings were used in making a SC classification model and to test the generalization ability of the model. Although the voice data consisted of five vowels and two repeated sentences per participant, we used only the sentence part for our study. A total of 21 features were extracted, and an advanced feature selection method—the least absolute shrinkage and selection operator (LASSO)—was applied to reduce the number of variables for classifier learning. A SC classification model was developed using multinomial logistic regression via LASSO.

**Results:**

We compared the proposed classification model to the previous study, which used both sentences and five vowels from the same patient’s group. The classification accuracies for the test set were 47.9% and 40.4% for male and female, respectively. Our result showed that the proposed method was superior to the previous study in that it required shorter voice recordings, is more applicable to practical use, and had better generalization performance.

**Conclusions:**

We proposed a practical SC classification method and showed that our model having fewer variables outperformed the model having many variables in the generalization test. We attempted to reduce the number of variables in two ways: 1) the initial number of candidate features was decreased by considering shorter voice recording, and 2) LASSO was introduced for reducing model complexity. The proposed method is suitable for an actual clinical environment. Moreover, we expect it to yield more stable results because of the model’s simplicity.

## Background

Sasang constitutional medicine (SCM) is a type of tailored, traditional Korean medicine. It divides human beings into four Sasang constitutional (SC) types—Tae-Yang (TY), Tae-Eum (TE), So-Yang (SY), So-Eum (SE)—according to their inherited characteristics, such as personality, appearance, susceptibility to particular diseases, and drug responses [1,2]. Determining one’s SC type is important to ensure proper treatment is performed.

Diagnosis using voice is an important part of SCM. The voice characteristics for each SC type, as referenced in the literature [3,4], and summarized in Table 1. The voice characteristics are described by linguistic form, which include utterance style and personality term. Since the linguistic representation can be understood differently, voice diagnosis in SCM is subjective and unreliable. Therefore, a quantitative interpretation explaining voice characteristics is required for standardizing diagnoses.

**Table 1:** Voice characteristics in each constitutional type

**Constitution**	**Voice characteristics**
TY	talkative, impatient, clear, influential, loud, resonant
SY	vigorous, clear, fruity, talkative, fast, hasty, illogical, impatient, high-pitched
TE	regular, taciturn, thick, loud, resonant, grave, dignified
SE	unstrained, artless, easy, sharp, not clear, not hoarse, still, calm, gentle, slow, low

Many attempts using computerized voice analysis methods for constitutional diagnosis have been made. Most studies excluded TY types since the number of TY types is too small to be analyzed statistically. As a result, SC diagnosis was treated as three-class classification problem. Various vocal features such as the pitch, frequency, formant, and energy of vocal signal were considered as quantitative features [5-7]. Park and Kim experimented with 71 people and found that there was a significant difference between SE and SY types in formant frequency and formant bandwidth [8]. Kim et al. developed a voice analysis system called the Phonetic System for Sasang Constitution-2004 and used it to study voices characteristics of 231 adult males [9] and 217 adult females [10]. Choi et al. used various features from a sentence utterance [11]. A total of 195 adult males took part in their study. Kang et al. used 144 vocal features from 473 people pronouncing five vowels and two repeated sentences [12]. They further developed a constitutional classification method [13] based on a support vector machine [14]. In their study, 32.2% of the voice data were classified into three types with 79.8% accuracy. Although many studies have tried to find a relationship between vocal features and constitution, these studies used only a limited number of subjects, and as a result, have not been successfully applied to larger datasets. To reflect common aspects of many SCM experts, a larger set of data, collected from 23 different oriental clinics, was established in the study by Do et al. [15]. More than 2,000 patients’ voice recordings were analyzed, and a classification model was proposed for the three SC types. The recording contents were five vowels and two repeated sentences, similar to the study by Kang et al. [12,13].

In this paper, we use the same voice data as in the previous study [15], since this is the largest dataset containing patient SC types as proved by herbal remedy [16]. Although the original voice data consists of five vowels and two repeated sentences recording, we only focus on the sentence part. It is important to note that both vowels and sentences data might be necessary for a full clinical examination including constitutional diagnosis, or diagnosis of a voice disorder [17] or vocal cord dysfunction [18]. In practice, a short recording is required for practical use for the u-health system and mobile phone applications.

The idea behind our approach is that a sentence, rather than vowels, can effectively represent a person’s voice characteristics. If a constitutional diagnosis model generated from only a sentence performs similarly or better than a model using both vowel and a sentence data, it is more appropriate for practical use. We propose a diagnosis model that consists of fewer vocal feature variables than the previous study [15]. The number of candidate feature variables is initially small since features from vowels are not considered. Moreover, feature selection based on the least absolute shrinkage and selection operator (LASSO) [19] was applied to reduce the number of variables in the classifier design. Experimental results show that the proposed method is not only practical, requiring shorter recording time and less variables to process, but also superior to the previous study in the generalization test.

## Methods

### Overview

**Figure 1:**
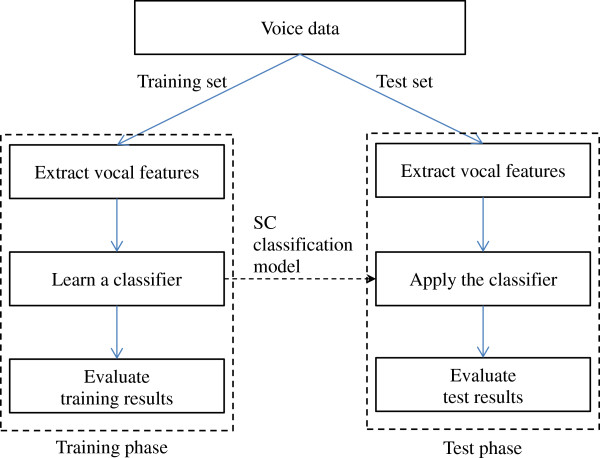
Overview of the SC classification process.

This study followed a typical statistical pattern classification framework. Figure 1 shows the flow chart of the SC classification process. Voice data was separated into two parts, the training set and the test set. In the training phase, vocal features were extracted from the training set and a SC classification model was acquired using the proposed LASSO-based method. Evaluation of the training results was performed to examine the fitting ability of the obtained model to the training set. In the test phase, the same procedure was applied to the test set. Generalization ability of the obtained classifier was evaluated using the test set.

### Voice data acquisition

Voice data were collected from 23 oriental medical clinics. Patients were examined and their SC type was determined by SCM practitioners having more than five years of clinical experience. A more detailed procedure of data collection is described by Song et al. [16]. We also collected face, body shape, and questionnaire data; however, only voice data were considered in this study.

Recording environment and procedure were strictly controlled by a standard operating procedure. Environmental noise was kept below 40 dB for low noise recording. To record, a Sound Blaster Live 24bit external soundcard and Sennheiser e-835 s microphone with a microphone stand were used. The distance between the patient and microphone was approximately 5 cm. Recordings were saved as wav files with a setting of mono 16bit integer and 44.1 kHz sampling frequency. Voice data consisted of five vowels (‘a,’ ‘e,’ ‘i,’ ‘o,’ and ‘u’) and two repeated sentences. The patient was asked to pronounce using their natural voice with the least amount of tension as possible. Each vowel was uttered at least 3 s. The sentence was uttered in the individual’s ordinary speed and tone. This process was approved by the Korea Institute of Oriental Medicine - Institutional Review Board (I-0910/02-001) and we obtained written informed consent from all study participants. In this paper, we excluded the vowel data and used only sentence data to diagnose a constitution.

### Vocal feature extraction

Vocal features were extracted using a C++ program combined with the hidden Markov model toolkit [20]. Vocal signals were divided into many windows based on the minimal time duration for feature extraction. The window size was 46.4 ms, which was mapped to 2^11^ samples in 44.1 kHz sampling frequency. Neighboring windows were overlapped by 50%. We used 2^n^ (n = 11) form for determining window size, since the exponential form does not require zero-padding in a fast Fourier transform [21].

We determined a candidate vocal feature pool including sF0 (average pitch), sFSTD (standard deviation of pitches), sI0 (average intensity), sISTD (standard deviation of intensities), and frequency-related features. A total of 21 features were extracted, and the description of each feature is shown in Table 2. Mel-frequency cepstral coefficients (MFCCs) are coefficients of the short-term power spectrum of a sound, based on a linear cosine transform of a log power spectrum on a nonlinear mel scale of frequency [22]. The mel scale approximates the human auditory system’s response more closely than linearly-spaced frequency bands. MFCCs are widely used in speech and speaker recognition systems [23]. We also included sSPD (reading speed) and sLPR, representing log power ratio over fixed frequency bands. Three frequency bands, 60 ~ 240 Hz, 240 ~ 960 Hz, and 960 ~ 3,840 Hz, were set for log power ratio features. For example, sLPR1 represented the log power ratio of the frequency range 60 ~ 240 Hz to 240 ~ 960 Hz. sLPR2 and sLPR3 are similarly defined in Table 2.

**Table 2:** Descriptions of vocal features

**Features**	**Descriptions**
sF0, sFSTD	Average pitch and standard deviation
sI0, sISTD	Average intensity and standard deviation
sMFCC0 ~ 12	13 Mel-frequency cepstral coefficients
sSPD	Reading speed for a sentence
sLPR1	Log power ratio (60 ~ 240 Hz/240 ~ 960 Hz)
sLPR2	Log power ratio (240 ~ 960 Hz/960 ~ 3840 Hz)
sLPR3	Log power ratio (60 ~ 240 Hz/960 ~ 3840 Hz)

All feature values are based on the averaged output of the two sentence utterances, which were repeated recordings of the same sentence.

### Data preprocessing

From the total number of 2,020 samples in the initial dataset, 55 cases involving individuals less than 15 years of age were excluded owing to their unstable acquisition of vocal measure. A total of 54 patients whose SC type was diagnosed as TY were also excluded because of its small sample size compared to the other three SC types. Since the measured vocal features, in general, showed non-linear fluctuation according to age, a process to reduce the effect of age was required. To eliminate the age effect to the vocal features, a standardization process, identical to that of Do et al.’s [15], was performed. All measured variables were standardized by using their moving averages and standard deviations derived from the samples within ±5 years of age for a specific age.

Prior to the moving average calculation, 11 outliers (five males and six females) were excluded by using the multivariate outlyingness measure and adjusted boxplot [24,25]. Furthermore, 31 influential cases (13 males and 18 females) were identified by influence measures [26] such as difference in fits (DFFITS), hat values, standardized residuals, and Cook’s distance. The influence measures are calculated from a binary logistic regression model for one vs. others (e.g., TE group vs. non-TE group). The influential cases were excluded from the dataset since their absence significantly changes the estimated coefficients in the regression model.

Finally, a total of 1,869 samples (658 males and 1,211 females) were used to train a gender specific SC classification model. To validate the performance of the trained model, 472 samples (165 males and 307 females) were used as a test set and with applying the same criteria for exclusion. Table 3 shows the distribution of SC types (except TY) according to gender and age for both the training and test sets. All measured vocal features were standardized with mean 0 and standard deviation 1. All analyses were performed by using the statistical package R-2.15.1 on the Windows platform.

**Table 3:** The distribution SC for both train and test set according to age and gender

	**Male**			**Female**		
	**TE**	**SE**	**SY**	**TE**	**SE**	**SY**
Train						
Age 15-19	†6 (27.3)	9 (40.9)	7 (31.8)	7 (30.4)	8 (34.8)	8 (34.8)
20-29	26 (37.7)	25 (36.2)	18 (26.1)	47 (29.0)	54 (33.3)	61 (37.7)
30-39	45 (37.8)	36 (30.3)	38 (31.9)	63 (26.9)	77 (32.9)	94 (40.2)
40-49	59 (42.1)	34 (24.3)	47 (33.6)	98 (34.9)	70 (24.9)	113 (40.2)
50-59	72 (47.7)	31 (20.5)	48 (31.8)	90 (36.7)	66 (26.9)	89 (36.3)
60-69	50 (46.3)	17 (15.7)	41 (38.0)	74 (42.5)	39 (22.4)	61 (35.1)
>70	26 (53.1)	8 (16.3)	15 (30.6)	45 (48.9)	26 (28.3)	21 (22.8)
ΌTotal	284 (43.2)	160 (24.3)	214 (32.5)	424 (35.0)	340 (28.1)	447 (36.9)
Test						
Age 15-19	2 (25.0)	6 (75.0)	-	3 (33.3)	2 (22.2)	4 (44.4)
20-29	2 (16.7)	5 (41.7)	5 (41.7)	9 (47.4)	7 (36.8)	3 (15.8)
30-39	8 (40.0)	5 (25)	7 (35)	18 (34.0)	23 (43.4)	12 (22.6)
40-49	16 (42.1)	9 (23.7)	13 (34.2)	27 (42.2)	20 (31.3)	17 (26.6)
50-59	19 (43.2)	6 (13.6)	19 (43.2)	30 (37.5)	18 (22.5)	32 (40.0)
60-69	12 (44.4)	3 (11.1)	12 (44.4)	27 (46.6)	14 (24.1)	17 (29.3)
>70	8 (50.0)	-	8 (50.0)	12 (50.0)	6 (25.0)	6 (25.0)
ΌTotal	67 (40.6)	34 (20.6)	64 (38.8)	126 (41.0)	90 (29.3)	91 (29.6)

†: N (%).

### Classification method

In the previous study conducted by Do et al., multinomial logistic regression (MLR) was applied to classify three SC types (TE, SE, and SY) using 88 vocal features. The classical MLR is a relatively good classifier for modeling the probability of membership of a class from the linear combination of the given features estimated by the ordinary least square (OLS) method. The OLS estimator, however, may be acquired with a large variance of coefficients and be impossible to solve if the dimension of explanatory features is too high or each of them is highly inter-correlated. These problems are referred to as over-fitting and multicollinearity, respectively. One well-known solutions to these problems is LASSO, which shrinks the variance of the coefficients and makes other coefficients zero [19].

As shown in Additional file 1 and Additional file 2, pairwise partial correlation coefficients for the obtained vocal features controlled for the age factor are significant in both male and female groups. Therefore, MLR via LASSO might be a better approach than OLS in terms of dealing with the multicollinearity problem and to acquire a stable and parsimonious model to classify SC types using vocal features.

We now give a brief description of the LASSO model considering the usual linear regression model. Let (*y*_1_, **x**_1_), …, (*y*_*n*_, **x**_*n*_) be paired observations where a response variable y∈ℝ and an explanatory vector of vocal features $x\in {\mathbb{R}}^{p}$. We estimate the *p* dimensional regression coefficient vector ***β*** by the regression function *E*(*y*|**x**) = *β*_0_ + **x**^*T*^***β***. The solution of the LASSO estimator ${\hat{\beta }}_{\mathrm{lasso}}\left(\lambda \right)$ can be obtained from the following formula,

(1) $$\left({\hat{\beta }}_{0},{\hat{\beta }}_{\mathrm{lasso}}\left(\lambda \right)\right)=\underset{\left\{\beta_{0},\beta \right\}\in {\mathbb{R}}^{p+1}}{\arg\;\text{min}}\left\{\sum_{i=1}^{N}{\left(y_{i}-\beta_{0}-\sum_{j=1}^{p}x_{\mathrm{ij}}\beta_{j}\right)}^{2}+\lambda \sum_{j=1}^{p}\left|\beta_{j}\right|\right\},$$

where ${\hat{\beta }}_{0}=\overset{-}{y}$, *β*_*j*_ is the *j*^th^ element of the vector ***β***, $\lambda \sum_{j=1}^{p}\left|\beta_{j}\right|$ is the penalty function, and *λ* is the regularization parameter for controlling the model complexity. The penalty function of LASSO performs the shrinkage of some of the regression coefficients to zero when *λ* is sufficiently large.

To build the classification model for SC types, coefficients for 21 vocal features were initially estimated via LASSO using the glmnet package implemented in the R software. The tuning parameter *λ* was selected from the result of 10-fold cross validation using the mean absolute error (MAE) to measure the risk of loss. The decision rule is to choose the largest log (*λ*) within one standard error of the minimum so-called “one standard error” rule [27].

The estimated response value or score, *η*_*k*_, corresponding to each SC type, where *k* = {1, 2, 3} represents the level of group category for SC (TE, SE, and SY, respectively), is simply expressed as

(2) $$\eta_{k}={\hat{\beta }}_{k0}+x^{T}{\hat{\beta }}_{k,\mathrm{lasso}}\left(\lambda '\right),$$

where $\left\{{\hat{\beta }}_{k0},\;{\hat{\beta }}_{k,\mathrm{lasso}}\left(\lambda^{'}\right)\right\}$ are estimated regression coefficients via LASSO for each group corresponding to *k*. Specifically, ${\hat{\beta }}_{k0}$ is the intercept term of the classification model for each SC type and ${\hat{\beta }}_{k,\mathrm{lasso}}\left(\lambda^{'}\right)$ is the *p* × 1 coefficient vector with respect to the tuning parameter *λ*^’^ as determined by the rule described above.

After acquisition of *η*_*k*_, the classical MLR model was applied to build the classification model. To do this, the score vector **η** = [*η*_1_, *η*_2_, *η*_3_] was used to classify the SC types using the VGAM package in the R software. The final model was adjusted for age. Letting **c** be the 1 × 4 concatenated vector of age and **η**, then the MLR models for logits with the reference category can be expressed as

(3) $$\log\left(\frac{\pi_{l}}{\pi_{k^{*}}}\right)=\gamma_{l0}+c^{T}\gamma_{l},$$

where {*γ*_*l*0_,  ***γ***_*l*_} are estimated regression coefficients for the group corresponding to *l* via classical MLR given **c**, *l* = {1, 2} ⊂ *k* is the index for TE and SE types, and the reference category k* = 3 represents the SY type. The estimated probability for being in each SC type *π*_*k*_ is given by:

(4) $$\pi_{k}=\frac{\exp\;\left(\gamma_{k0}+c^{T}\gamma_{k}\right)}{\sum_{k=1}^{3}\exp\left(\gamma_{k0}+c^{T}\gamma_{k}\right)}\;.$$

Finally, let *g* ∈ {1, 2, 3} be the predicted SC types. The classification is done by choosing the SC type that has the maximum probability, given by:

(5) $$g=\underset{k\in \left\{1,2,3\right\}}{\arg\;\max}\left(\pi_{k}\right).$$

## Results

### Classification model using MLR via LASSO

**Figure 2:**
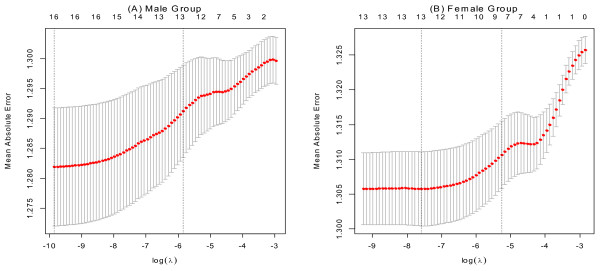
**Ten-fold cross validation results for training sets corresponding to each gender group.** Panel **(A)** represents the result of the male group and panel **(B)** represents the female group. For both panels, the average cross-validated curves (red dots) with a one standard deviation band are shown. The axis on the left shows the MAE, the axis on the bottom shows the value of log (*λ*), and the vertical line on the left is located at the minimum cross validation error while the right vertical line is located at the maximum value of log (*λ*) within 1 standard error (S.E) of the minimum. The axis on the top represents the average cross-validated number of selected variables corresponding to log (*λ*).

To set the tuning parameter λ in (1), 10-fold cross validation using MAE was performed. Figure 2 shows the MAE distribution along with the change of log (λ). As the largest value of log (*λ*) within one standard error of the minimum average MAE, we picked log(*λ*^'^)_*male*_ to be 5.844 for male and log(*λ*^'^)_*female*_ to be − 5.260  for female.

Table 4 shows the results of coefficients $\left({\hat{\beta }}_{0},{\hat{\beta }}_{\mathrm{lasso}}\left(\lambda \right)\right)$ in (1) for each SC type. Not all vocal features were selected. The selected features varied in each SC type. For male, 7, 12, and 13 features were selected for TE, SE, and SY, respectively. For female, 11, 8, and 7 features were selected for TE, SE and SY, respectively.

**Table 4:** Result of estimated coefficients for vocal features for each SC type using LASSO

	**Male**			**Female**		
**Features**	${\hat{\beta }}_{1,\mathrm{lasso}}\left(\lambda^{'}\right)$	${\hat{\beta }}_{2,\mathrm{lasso}}\left(\lambda^{'}\right)$	${\hat{\beta }}_{3,\mathrm{lasso}}\left(\lambda^{'}\right)$	${\hat{\beta }}_{1,\mathrm{lasso}}\left(\lambda^{'}\right)$	${\hat{\beta }}_{2,\mathrm{lasso}}\left(\lambda^{'}\right)$	${\hat{\beta }}_{3,\mathrm{lasso}}\left(\lambda^{'}\right)$
${\hat{\beta }}_{k0}\;$	0.310	−0.309	0.000	0.042	−0.157	0.116
sF0	0.081			−0.112	0.015	.
sFSTD		−0.209	0.168		−0.048	.
sI0		0.159	−0.212	−0.071	0.062	.
sISTD	0.107		−0.019			−0.040
sSPD	−0.004		0.081	0.048		−0.015
sLPR1			0.060		0.017	−0.004
sLPR2	0.197		−0.126	0.049		−0.067
sLPR3		−0.031				−0.012
sMFCC0		−0.007	0.207			
sMFCC1	−0.213	0.065		−0.027		0.081
sMFCC2		−0.068	0.218	−0.071	0.001	
sMFCC3	0.238		−0.127	−0.083	0.032	
sMFCC4	0.124					0.020
sMFCC5		0.096	−0.105	0.012	−0.010	
sMFCC6		0.170	−0.028	−0.035		
sMFCC7		0.126	−0.067	−0.126	0.016	
sMFCC8		0.032		0.263		
sMFCC9		−0.119		0.215	−0.038	
sMFCC10		−0.099	0.146			
sMFCC11	0.109	−0.074		−0.032		0.027
sMFCC12			0.046	−0.099		0.126

$${\hat{\beta }}_{1,\mathrm{lasso}}\left(\lambda^{'}\right)$$ , ${\hat{\beta }}_{2,\mathrm{lasso}}\left(\lambda^{'}\right)$, and ${\hat{\beta }}_{3,\mathrm{lasso}}\left(\lambda^{'}\right)$ represent result of estimated coefficients via LASSO for each SC group, TE, SE, and SY, respectively, where $\lambda^{'}=e^{\log\left(\lambda^{'}\right)}$, chosen from the result of ten-fold cross validation. The shrinkage coefficients to zero are annotated with ‘.’.

Since the group of SY was defined as a reference class, two MLR models were obtained for the TE and SE groups. The parameter **γ**, its standard error, and Wald’s Z are summarized in Table 5.

**Table 5:** Final result of MLR for the classification of SC using age and score η estimated by LASSO

		**Male**			**Female**		
		**γ**_***l,***_	**S.E.**	**Wald’s Z**	**γ**_***l,***_	**S.E.**	**Wald’s Z**
TE (*l* = 1)	γ_0_	−0.284	0.341	−0.834	−0.498	0.275	−1.810
	Age	0.004	0.006	0.691	0.011	0.005	2.374
	*η*_1_	1.254	0.442	2.838	1.116	0.234	4.776
	*η*_2_	0.001	0.312	0.004	0.105	0.823	0.127
	*η*_3_	−1.222	0.339	−3.603	−1.297	0.521	−2.492
SE (*l* = 2)	γ_0_	0.754	0.375	2.014	0.251	0.280	0.897
	Age	−0.017	0.007	−2.331	−0.003	0.005	−0.561
	η_1_	0.241	0.519	0.464	−0.087	0.240	−0.361
	η_2_	1.078	0.372	2.897	1.453	0.863	1.683
	η_3_	−1.176	0.380	−3.094	−1.464	0.544	−2.689

Reference category: SY (k* = 3) S.E: standard error of *γ*_*l, m*_ , where *m* = 1,…,4 , indicating the *m*^*th*^ element of estimated coefficient vector **γ**_*l*_, Wald’s Z: Wald’s statistics to test the null hypothesis *H*_0_ : *γ*_*l*,*m*_ = 0.

### Classification results

To make a fair comparison, we used a common set of samples between our study and the previous study conducted by Do et al. [15]. Because the initial feature pool, outliers, and influential cases were different in both studies, the remaining sets of samples after data preprocessing were not identical. Therefore, an intersection of the data in both studies was required. A total of 1,692 samples (593 males and 1,099 females) were used as an intersection training set; note that in total, 1,869 samples (658 males and 1,211 females) were used to train the SC classification model. Similarly, 472 samples (165 males and 307 females) were used as an intersection test set.

In [15], MLR via OLS was applied to classify three SC types using 88 vocal features from five vowels and one sentence. Our methods incorporated MLR via LASSO and used 21 features from the sentence only. The comparison results are shown in Table 6. The classification accuracies for the training set were 46.5% and 44.0% for male and female, respectively, which were worse than those in Do et al.’s study. However, the accuracies for the test set, 47.9% and 40.4% for male and female, respectively, were better than those in Do et al.’s study. The proposed method did not have a significant difference between accuracies of the training and test sets, which clearly showed the LASSO dealt well with multicollinearity. The accuracies of the training set in Do et al. was higher than ours, however, there were significant drops in accuracies of the test set. Since they used a larger number of features, it may have caused over-fitting to the training set. The results showed that the generalization ability of the proposed model was better than that of the previous study.

**Table 6:** Comparative results between the model in Do et al. and the proposed model via LASSO

			**Male**					**Female**				
			**Predicted SC**				**Sensitivity**	**Predicted SC**				**Sensitivity**
			**TE**	**SE**	**SY**	**Total**		**TE**	**SE**	**SY**	**Total**	
**Do et al.**												
		**TE**	185	31	43	259	71.4%	204	34	150	388	52.6%
Train	**True SC**	**SE**	52	62	33	147	42.2%	95	63	144	302	20.9%
		**SY**	84	26	77	187	41.2%	137	43	229	409	56.0%
		**Total**	**321**	**119**	**153**	**593**		**436**	**140**	**523**	**1099**	
		Accuracy	54.6%					45.1%				
		**TE**	46	6	15	67	68.7%	51	15	60	126	40.5%
Test	**True SC**	**SE**	14	4	16	34	11.8%	32	20	38	90	22.2%
		**SY**	37	12	15	64	23.4%	37	11	43	91	47.3%
		**Total**	**97**	**22**	**46**	**165**		**120**	**46**	**141**	**307**	
		Accuracy	39.4%					37.1%				
**Proposed**												
		**TE**	184	22	53	259	71.0%	212	20	156	388	54.6%
Train	**True SC**	**SE**	86	30	31	147	20.4%	119	26	157	302	8.6%
		**SY**	110	15	62	187	33.2%	137	26	246	409	60.1%
		**Total**	**380**	**67**	**146**	**593**		**468**	**72**	**559**	**1099**	
		Accuracy	46.5%					44.0%				
		**TE**	49	2	16	67	73.1%	70	7	49	126	55.6%
Test	**True SC**	**SE**	16	10	8	34	29.4%	30	7	53	90	7.8%
		**SY**	41	3	20	64	31.3%	43	1	47	91	51.6%
		**Total**	**106**	**15**	**44**	**165**		**143**	**15**	**149**	**307**	
		Accuracy	47.9%					40.4%				

## Discussion and conclusions

In this study, a practical method for Sasang constitutional diagnosis was proposed. The proposed method was developed with a large number of training data and tested using data not included in the training set. The classification accuracies of the test results were 47.9% and 40.4% for male and female, respectively. We compared the diagnosis accuracy to a previous study using the same data. Although the proposed method had lower results for the training data, it obtained higher test results for both male and female test data. It should be noted that our approach had better generalization ability, even requiring shorter recording content, which is important for practical use.

In contrast to the previous study, which used 88 features from five vowels and one sentence, the proposed method used 21 features from the sentence only. We showed that our model having few variables outperforms one having many variables in a generalization test. A large number of variables usually cause over-fitting problems by increasing model complexity and as a result, the trained model tends to be sensitive to noise samples. We attempted to reduce the number of variables in two ways. First, the initial number of candidate features was decreased by considering only sentence information. Many of the features were extracted from five vowels in the previous study; however, they did not seem to play an important role in the diagnosis model.

Second, LASSO was introduced for reducing multicollinearity. Pairwise partial correlation coefficients in vocal features were examined to confirm multicollinearity. LASSO selected the proper numbers of variables so that not all of the 21 initial candidate features were used.

Although the diagnosis using voice does not have high accuracy by itself, it is helpful when used in conjunction with other diagnosis methods such as face, body shape, and a questionnaire. As of now, we are suffering from noisy features from the vowels; however, we still need to deal with the information from vowels and improve the diagnosis accuracy by combining the features from both vowels and sentence.

The proposed method is suitable in an actual clinical environment where patients might not pronounce a long recording content. Aged people especially have difficulty in uttering vowels for a long period of time. Our approach is also applicable to many voice-based diagnoses such as voice disorder detection, vocal cord dysfunction, and constitutional health diagnosis, expecting more stable results because of the simplicity of the model.

There are, however, some limitations of the proposed method. The classification model is trained by using a predefined sentence, and the same sentence must be pronounced during the test phase. If the recording content slightly changes during the test phase, the corresponding results are not acceptable. This can be problematic when extended to other languages. On the other hand, vowels have the advantage of being applied to other languages since vowels can be pronounced similarly among different languages. A short recording content may not represent patients’ vocal characteristics correctly. Although subjects are asked to pronounce in their ordinary tone without tension, some are nervous during the actual recording resulting in their recordings being different to their ordinary speech. An advanced voice analysis method, capable of dealing with more natural talking, is required for future research.

## Abbreviations

SCM: Sasang constitutional medicine; SC: Sasang constitution; TY: Tae-Yang; TE: Tae-Eum; SY: So-Yang; SE: So-Eum; LASSO: Least absolute shrinkage and selection operator; MFCC: Mel-frequency cepstral coefficients; DFFITS: Difference in fits; MLR: Multinomial logistic regression; OLS: Ordinary least square; MAE: Mean absolute error.

## Competing interests

The authors hold and are currently applying for patents relating to the content of the manuscript.

## Authors’ contributions

JSJ conceived the idea, designed the experiments and drafted the manuscript. BK performed the statistical analysis and interpretation of data. YSK carried out vocal feature extraction. JN participated in the statistical analysis and compared to the previous study. KHK participated in extracting vocal features. JYK conceived the study, and helped to draft the manuscript. All authors read and approved the final manuscript.

## Pre-publication history

The pre-publication history for this paper can be accessed here:

http://www.biomedcentral.com/1472-6882/13/307/prepub

## Supplementary Material

Additional file 1: Table S1 Pairwise partial correlation coefficients for vocal features in the male group controlled for age. Click here for file

Additional file 2: Table S2 Pairwise partial correlation coefficients for vocal features in the female group controlled for age. Click here for file
