# Supplementary material for: A practical approach to Sasang constitutional diagnosis using vocal features
Source: BMC Complement Altern Med. 2013 Nov 7;13:307. doi: 10.1186/1472-6882-13-307 (PMC4226254; doi:10.1186/1472-6882-13-307)
Supplement: Additional file 2: Table S2 — Pairwise partial correlation coefficients for vocal features in the female group controlled for age. [file 1472-6882-13-307-S2.pdf]

Table S2. Pairwise partial correlation coefficients for vocal features in the female group controlled for age

	sF0	sFSTD	sI0	sISTD	sSPD	sLPR1	sLPR2	sLPR3	sMFCC0	sMFCC1	sMFCC2	sMFCC3	sMFCC4	sMFCC5	sMFCC6	sMFCC7	sMFCC8	sMFCC9	sMFCC10	sMFCC11
sFSTD	0.45***																			
sI0	0.01	0.02																		
sISTD	0.30***	0.32***	0.29***																	
sSPD	0.07*	0.17***	0.08**	0.14***																
sLPR1	-0.50***	-0.32***	0.03	-0.28***	-0.09**															
sLPR2	-0.05	-0.07*	0.15***	0.11***	-0.11***	0.11***														
sLPR3	-0.40***	-0.28***	0.12***	-0.15***	-0.14***	0.81***	0.68***													
sMFCC0	0.01	0.01	0.93***	0.24***	-0.05	-0.1***	-0.03	-0.09**												
sMFCC1	-0.37***	-0.24***	0.25***	0.02	-0.26***	0.33***	0.67***	0.65***	0.07*											
sMFCC2	-0.58***	-0.29***	-0.01	-0.31***	0.01	0.7***	0.2***	0.64***	-0.11***	0.14***										
sMFCC3	-0.31***	-0.13***	0.03	-0.06*	0.04	0.35***	-0.29***	0.09**	-0.03	0.2***	0.04									
sMFCC4	-0.48***	-0.21***	-0.02	-0.23***	0.00	0.48***	0.05	0.39***	-0.05	0.05	0.64***	-0.2***								
sMFCC5	-0.18***	-0.06*	0.09**	0.01	0.07*	0.14***	-0.12***	0.03	0.06*	0.04	0.03	0.5***	-0.33***							
sMFCC6	-0.16***	0.03	-0.01	-0.10***	0.18***	0.08**	-0.08**	0.02	-0.01	-0.17***	0.25***	-0.11***	0.47***	-0.27***						
sMFCC7	-0.24***	-0.1***	0.00	0.05	0.07*	0.1***	0.1***	0.14***	-0.07**	0.25***	0.11***	0.15***	0.07*	0.31***	-0.42***					
sMFCC8	-0.05	0.04	-0.10***	-0.05	0.07*	-0.18***	-0.15***	-0.23***	-0.05	-0.2***	-0.01	0.09**	0.01	-0.01	0.49***	-0.52***				
sMFCC9	-0.21***	-0.09**	-0.04	0.08**	0.02	-0.04	0.22***	0.10***	-0.06*	0.19***	0.1***	-0.07*	0.14***	0.11***	-0.13***	0.47***	-0.37***			
sMFCC10	-0.09**	-0.06	-0.15***	-0.09**	-0.01	-0.13***	-0.19***	-0.22***	-0.06*	-0.21***	0.01	0.04	0.07*	-0.06*	0.08**	-0.17***	0.4***	-0.39***		
sMFCC11	-0.15***	0.04	-0.02	0.15***	0.12***	0.03	0.08**	0.07*	-0.06*	0.1***	0.09**	0.02	0.20***	0.00	0.16***	0.14***	-0.06	0.50***	-0.41***	
sMFCC12	0.02	0.08**	-0.06*	-0.05	0.11***	-0.01	-0.17***	-0.11***	-0.05	-0.2***	0.09**	0.12***	-0.14***	0.23***	-0.07*	0.06*	0.04	-0.08**	0.3***	-0.27***

The number of asterisks represents the magnitude of significance for p-values to test the null hypothesis $H_0: \rho_{(x_i x_j|age)} = 0,\ i, j = 1, \ldots, p$, where p is the total number of features; * (p<0.05), ** (p<0.01), *** (p<0.001), respectively.
